# Sustainable Diets and Cancer: a Systematic Review

**DOI:** 10.1007/s13668-022-00442-z

**Published:** 2022-11-21

**Authors:** Nena Karavasiloglou, Sarah T. Pannen, Carmen Jochem, Tilman Kuhn, Sabine Rohrmann

**Affiliations:** 1 Division of Chronic Disease Epidemiology, Epidemiology, Biostatistics and Prevention Institute, University of Zurich, Hirschengraben 84, Zurich, CH-8001 Switzerland; 2 Cancer Registry of the Cantons of Zurich, Zug, Schaffhausen and Schwyz, Institute of Pathology and Molecular Pathology, University Hospital Zurich, Switzerland, Vogelsangstrasse 10, Zurich, CH-8091 Switzerland; 3 Department of Epidemiology and Preventive Medicine, University of Regensburg, Franz-Josef-Strauß-Allee 11, Regensburg, D-93053 Germany; 4 Institute for Global Food Security, Queen’s University Belfast, 19 Chlorine Gardens, Belfast, UK; 5 Heidelberg Institute of Global Health (HIGH), Medical Faculty and University Hospital, Heidelberg University, Im Neuenheimer Feld 130.3, Heidelberg, D-69120 Germany

**Keywords:** Sustainability, Diet, Cancer, Risk, Mortality, Review

## Abstract

***Purpose of Review*:**

This review aimed to investigate the association of sustainable diets in relation to cancer risk, cancer recurrence, and cancer-specific mortality in adults.

***Recent Findings*:**

More than 500 articles were initially identified. Nine articles were eligible for inclusion, presenting data from 8 prospective cohort studies, conducted in Europe and the USA. The sustainability indicators investigated were greenhouse gas emissions, food biodiversity, land use, exposure to pesticides or organic food consumption, and the EAT-Lancet diet. One study reported a sustainability index that combined multiple sustainability indicators. A modest inverse association between higher adherence to sustainable diets and cancer incidence or cancer mortality was observed in most studies.

***Summary*:**

While sustainable diets may decrease cancer risk or mortality, the reviewed studies were heterogeneous regarding sustainability indicators and cancer outcomes. A common definition of dietary sustainability would facilitate better generalization of future research findings. Also, studies among non-western populations are needed.

**Supplementary Information:**

The online version contains supplementary material available at 10.1007/s13668-022-00442-z.

## Introduction

Studies suggest that food systems are important contributors to the global environmental crisis. For example, food systems are responsible for approximately one-third of greenhouse gas emissions [1]. While a growing body of evidence highlights the unsustainable nature of our current food systems, there is no universally agreed-upon definition of what constitutes an environmentally sustainable diet. Recently, the Food and Agriculture Organization of the United Nations (FAO) proposed the following definition: “Sustainable diets are those diets with low environmental impacts which contribute to food and nutrition security and to healthy life for present and future generations. Sustainable diets are protective and respectful of biodiversity and ecosystems, culturally acceptable, accessible, economically fair and affordable; nutritionally adequate, safe and healthy; while optimizing natural and human resources” [2]. Even before FAO proposed a definition, most scientists considered dietary patterns including a high proportion of plant-based foods as sustainable. The common denominator in all plant-based dietary patterns is a conscious effort to reduce or entirely exclude animal product consumption from one’s diet. However, these patterns include a vastly diverse range of products that can be consumed, resulting in different profiles of dietary intake.

In an effort to assess the sustainability of different diets or dietary patterns, various research groups have used different indices or measures. While their approaches differ considerably in terms of the diet assessed, the method used to assess the dietary intake, and the assessment of sustainability (e.g., focusing on specific indicators like greenhouse gas emissions or biodiversity versus creating indices that combine more than one sustainability assessment), most conclude that closely following sustainable diets might be associated with favorable health outcomes in terms of non-communicable diseases.

Globally in 2020, an estimated 19.3 million incident cancer cases were diagnosed, whereas almost 10.0 million people died from cancer [3]. Diet may play a crucial role in cancer risk [4–7], and scientific evidence suggests that dietary patterns mainly based on the consumption of plant foods are associated with a lower risk of many non-communicable diseases, including some types of cancer [8]. However, a systematic assessment of sustainable dietary patterns and cancer outcomes is lacking. Thus, we carried out a systematic review on sustainable diets in relation to cancer risk, cancer recurrence, and cancer-specific mortality in adult populations.

## Methods

Our systematic review was conducted according to the Preferred Reporting Items for Systematic Reviews and Meta-analyses (PRISMA) guidelines. The review was registered with PROSPERO under number CRD42022304761.

### Eligibility Criteria

We posed no time restrictions relating to the year of publication, language, or geographical location. Studies that investigated the association between adherence to sustainable diets and cancer risk as well as major outcomes among cancer patients (e.g., recurrence, cancer-specific mortality, all-cause mortality) among adults and reported effect estimates were included. Sustainable diets were defined as diets that are assessed using widely available sustainability indices. Traditional diets (e.g., the Mediterranean diet, vegan, or vegetarian diets), which are mainly plant-based and have been previously suggested as sustainable, were not included unless their sustainability was assessed by some specific measure or index.

Studies were excluded if they were duplicates, dealt with other topics, did not include an assessment of the sustainability of the diet, or reported on health outcomes other than the cancer-related outcomes defined above. Studies dealing with underaged populations or exclusively pregnant women, as well as those that focused on animals or cell cultures, were also excluded.

Prospective observational studies, including cohort, nested case–control, case-cohort, and randomized controlled trials, published in peer-reviewed journals were included. Existing reviews or meta-analyses, as well as conference abstracts, comments, letters, and opinions were excluded.

### Selection of Eligible Studies

We searched PubMed and EMBASE for eligible studies without restrictions on publication year using predefined search terms (Supplementary information). The search was conducted in early December 2021, and the screening process was conducted using the CADIMA web tool [9, 10].

Two researchers (NK, SP) screened all articles’ titles and abstracts against eligibility criteria to identify relevant studies (100% overlap between the researchers). If during the title and abstract screening process the result was not clear, we screened the full-text of the article. The same reviewers screened the studies in the full-text screening phase (100% overlap between the researchers).

### Data Extraction

After full-text screening, data from the included articles were extracted in a customized Microsoft Excel sheet. Multiple publications of the same study were only considered if they significantly differed from each other (either in the assessment of sustainability or the study outcome). The extraction included the name of the first author, year of publication, study name, study design, country, and time of data collection. We additionally extracted data on the original sample size, the total number of events (overall, and/or by cancer type; depending on availability), the sustainability assessment method, the confounders considered, and the reported effect estimate.

### Risk of Bias Assessment

To assess bias across the included studies, we used the Risk of Bias in Non-randomized Studies of Interventions (ROBINS-I) tool from Cochrane, adapted for observational studies where needed (Risk of Bias in Non-randomized Studies of Exposures, ROBINS-E), as described by Schwingshackl et al. [11]. For the assessment of the confounding domain, we pre-specified the following adjustment factors: age, sex, socio-economic status, smoking status, alcohol consumption, physical activity, obesity, height, reproductive factors, and energy intake. The *robvis* tool was used to visualize our Risk of Bias Assessment [12]. The certainty of the evidence was evaluated using GRADE (Grading of Recommendations Assessment, Development, and Evaluation, as described by Schwingshackl et al. [11], and the GRADE working group https://www.gradeworkinggroup.org/). The risk of bias and certainty of the evidence assessments was carried out by two researchers (TK, SR). In case of disagreements between the two authors, these were resolved with discussion between authors.

## Results

**Fig. 1:**
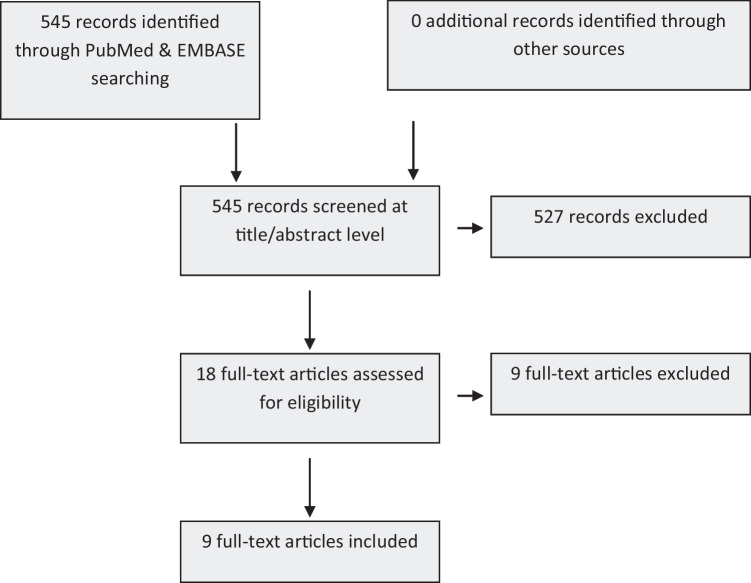
Flow diagram detailing the article selection process

Our search identified more than 500 records. After duplicate removal and the initial assessment at the level of title and abstract, the full-text screening identified 9 eligible articles for inclusion (Fig. 1). The eligible articles presented data from 8 prospective cohort studies, conducted in Europe and the USA. The European studies included multiple publications from the European Prospective Investigation into Cancer and Nutrition (EPIC, multiple countries included versus country-specific) as well as the NutriNet-Santé cohort.

The sustainability indicators investigated in the identified studies could be categorized into greenhouse gas emissions (2 studies), food biodiversity (1 study), land use (1 study), exposure to pesticides or organic food consumption (4 studies), the EAT-Lancet diet (1 study), and a sustainability index encompassing multiple sustainability indicators (1 study; Supplementary Table 1).

The incidence of cancer (overall, or for selected cancer sites) was the investigated outcome in seven articles (Table 1), while cancer-specific mortality was investigated in three (one eligible article investigated both cancer incidence and mortality; Table 2).

**Table 1:** Study characteristics and effect estimates from cohort studies on the association between sustainable diets and cancer incidence

**First author, year of publication**	**Study name, country**	**Sex, mean age at study entry**	**Sample size**	**Outcome**	**Number of cancer cases**	**Effect estimate, adjusted**	**Adjusting factors**
González et al., 2020 [8]	EPIC-Spain, Spain	62.3% women, 49.3 years	40,621	Cancer incidence	4457	HR for total cancer: 1.031 (95% CI: 0.998–1.065) for 1-unit increase in GHG emissions	Sex; stratified by Spanish center and age at recruitment
Laine et al., 2021 [13]	EPIC, 9 European countries	71% women, 52 years	443,991	Cancer incidence	58,925	HR per quartiles of GHGs emissions _Q4__vs.__Q1_: 1.11 (95% CI: 1.09–1.14) HR per land use contributions _Q4__vs.__Q1_: 1.13 (95% CI: 1.10–1.15) In site-specific analyses, there was a positive association between GHGs emissions and cancers of the bladder, renal pelvis, ureter, and other urinary organs, breast, colorectum, esophagus, kidney, larynx, lung, skin melanoma, stomach, and thyroid. There was also a positive association between land use and cancers of the brain and CNS, bladder, renal pelvis, ureter and other urinary organs, breast, colorectum, esophagus, kidney, larynx, liver, lung, myeloma, pancreas, prostate, skin melanoma, stomach, and thyroid	Age at recruitment, marital status, education, physical activity, smoking status, BMI
Sandoval-Insausti et al., 2021 [14]	Nurses’ Health Study, Nurses’ Health Study II and Health Professionals Follow-up Study, USA	Sex and mean age not given	Not given	Cancer incidence	23,678	HRs of total cancer associated with a 1 serving/day increase in intake: 1.01 (95% CI: 0.99–1.02) for low-pesticide-residue FVs 0.99 (95% CI: 0.97–1.01) for high pesticide residue fruits and vegetables High-pesticide-residue fruit and vegetable intake HR_Q5__vs.Q1_: 1.00 (95% CI: 0.95–1.05), *p*-trend 0.77 Low-pesticide-residue fruit and vegetable intake HR_Q5vs.Q1_: 0.99 (95% CI: 0.95–1.04), *p*-trend 0.74 In site-specific analyses, no association between intake of high-pesticide-residue FVs or low-pesticide-residue FV and risk of any sites was seen	Age, height, BMI, ethnicity, physical activity, family history of cancer, physical examination in the past 2 years, history of colonoscopy or sigmoidoscopy, mammography in the past 2 years, prostate-specific antigen testing in the past 2 years, number of pack-years among ever smokers, postmenopausal hormone use, current multivitamin use, regular aspirin use, total energy intake, alcohol intake, and Alternate Healthy Eating Index score excluding criteria for intake of fruits and vegetables and alcohol. Additionally adjusted for intakes fruits and vegetables with undetermined residues and low-pesticide-residue fruits and vegetables, or high-pesticide-residue fruits and vegetables, respectively
Rebouillat et al., 2021 [15]	NutriNet-Santé, France	100% women, 60.5 years	13,149	Cancer incidence	169	NMF component 1 HR_Q5__vs.__Q1_: 1.77 (95% CI: 1.07–2.91), *p*-trend: 0.08; NMF Component 2 HR_Q5__vs.__Q1_: 0.99 (95% CI: 0.61–1.62), *p*-trend: 0.37; NMF component 3 HR_Q5__vs.__Q1_: 0.59 (95% CI: 0.36–0.98), p-trend: 0.01; NMF component 4 HR_Q5__vs.__Q1_: 0.66 (95% CI: 0.39–1.12), *p*-trend: 0.13; There was a significant negative association between NMF Component 3 (low synthetic-pesticide-exposure profile) and postmenopausal breast cancer risk Stratified analysis among women with BMI > 25 kg/m^2^ found a positive association between NMF component 1 and postmenopausal breast cancer risk: HR_Q5__vs.__Q1_: 4.13 (95% CI: 1.50–11.44), *p*-trend: 0.006)	Age, smoking practices, educational level, physical activity, alcohol intake, alcohol-free energy intake, BMI, height, family history of cancer, menopausal treatment and parity, a score for overall quality of the diet based on the level of adherence to 2017 French dietary guidelines, a provegetarian score, the percentage of ultra-processed foods in the diets, or residing in an agricultural area
Seconda et al., 2020 [18]	NutriNet-Santé, France	Sex distribution by quartiles of Sustainable Diet Index (SDI): Q1: 76.2%, Q2: 74.5%, Q3: 78.6% and Q4: 74.5% female, respectively. Mean age not given	25,589	Cancer incidence	483 (138 breast, 78 prostate, 46 colorectal, 45 skin, 22 lung and 154 other cancers)	All cancers: HR SDI _Q4__vs.__Q1_: 0.56 (95% CI: 0.41–0.77), p-trend: 0.0002 Inverse associations were also reported for breast, and smoking-related cancer (model without adjustment for BMI)	Age, sex, scholar graduation, smoking status, income by household units, occupational status, alcohol status, family history of cancer, physical activity, energy consumption, height, BMI and (for women) parity, postmenopausal status, use of hormonal treatment for menopause, and use of oral contraception
Baudry et al., 2018 [16]	Nutrinet-Santé, France	78.0% women, 44.2 years	68,946	Cancer incidence	1340 (459 breast, 180 prostate, 135 skin, 99 colorectal, 47 non-Hodgkin lymphomas, and 15 other lymphomas)	All cancers HR OFS _Q4__vs.__Q1_: 0.76 (95% CI: 0.64–0.90), *p*-trend: 0.003 HR per 5-point increase (model 3): 0.93 (95% CI: 0.89–0.97) In site-specific analyses, a higher OFS was associated with a reduced risk of postmenopausal breast cancer, non-Hodgkin lymphoma, and all lymphomas. No statistically significant association was observed for overall breast, premenopausal breast, prostate, colorectal, and skin cancer	Age, sex, month of inclusion, occupational status, educational level, marital status, monthly income per household unit, physical activity, smoking status, alcohol intake, family history of cancer, BMI, height, energy intake, a score for overall quality of the diet reflecting adherence to the official French nutritional guidelines, fiber intake, processed meat intake and red meat intake, ultraprocessed food consumption, fruit and vegetable consumption, and dietary patterns extracted by principal component analysis and (for women) parity, postmenopausal status, use of hormonal treatment for menopause, and use of oral contraception
Bradbury et al., 2014 [17]	Million Women Study, UK	100% women	623,080	Cancer incidence	53,769	RR “Sometimes OF” vs. “Never OF”: 1.03 (95% group-specific CI: 1.01–1.04) RR “Usually/always OF” vs. “Never OF”: 1.03 (95% group-specific CI: 1.00–1.06) In site-specific analyses, more frequent consumption of was associated with higher risk for breast cancer, and lower risk for non-Hodgkin lymphoma	Age, region, deprivation, smoking, BMI, physical activity, alcohol intake, height, parity and age at first birth, fiber intake, type of meat eaten

The adjustment factors and the results are reported for the fully adjusted model, unless otherwise specified. When categorical results are reported, only the extreme categories are shown

*BMI* body mass index, *CI* confidence interval, *CNS* central nervous system, *EPIC* European Prospective Investigation into Cancer and Nutrition, *FV* fruit and vegetables, *GHG* greenhouse gas, *HR* hazard ratio, *NMF* non-negative matrix factorization, *OF* organic food, *OFS* organic food score, *RR* relative risk, *SDI* Sustainable Diet Index

**Table 2:** Study characteristics and effect estimates from cohort studies on the association between sustainable diets and cancer-specific mortality

**First author, year of publication**	**Study name, country**	**Sex, mean age at study entry**	**Sample size**	**Outcome**	**Number of cancer-related deaths**	**Effect estimate (95% CI), adjusted**	**Adjusting factors**
Hanley-Cook et al., 2021 [19]	EPIC, 9 European countries	71% women, 51 years	451,390	Cancer-specific mortality	19,284	HR per 10-species increment DSR: 0.93 (95% CI: 0.92–0.95) HR per quartiles of DSR, species per year: _Q5__vs.__Q1_: 0.75 (95% CI: 0.69–0.82), *p*-trend: < 0.001	Stratified for center, age at recruitment, and sex and adjusted for baseline alcohol intake, physical activity, marital status, smoking status and intensity of smoking, educational level, baseline energy intake, baseline fiber intake, baseline red and processed meat consumption, and an 18-point Mediterranean diet score
Laine et al., 2021 [13]	EPIC, 10 European countries	71% women, 52 years	443,991	Cancer-specific mortality	14,095	HR per quartiles of GHG emissions _Q4__vs.__Q1_: 1.16 (95% CI: 1.10–1.22) HR per land use contributions _Q4__vs.__Q1_: 1.21 (95% CI: 1.16–1.27)	Age at recruitment, marital status, education, physical activity, smoking status, BMI, and country
Stubbendorff et al., 2021 [21]	Malmö Diet and Cancer Study, Sweden	61.8% women, Mean age not given, only ranges reported in the paper	22,421	Cancer-specific mortality	2655	HR per EAT-Lancet score: _≥23__vs.__≤13_: 0.76 (95% CI: 0.63–0.92) Women HR EAT-Lancet score _≥__23__or_ _vs.__≤13_: 0.81 (95% CI: 0.62–1.05) Men HR EAT-Lancet score _≥__23__or_ _vs.__≤13_: 0.64 (95% CI: 0.45–0.90)	Age, sex, dietary assessment version, season, energy intake, leisure-time physical activity, smoking habits, alcohol consumption, educational level, and BMI

The adjustment factors and the results are reported for the fully adjusted model, unless otherwise specified. When categorical results are reported, only the extreme categories are shown

*BMI* body mass index, *CI* confidence interval, *DSR* dietary species richness, *EPIC* European Prospective Investigation into Cancer and Nutrition; GHG: Greenhouse Gas, *HR* hazard ratio

### Cancer Incidence

#### Greenhouse Gas Emissions

Two studies reported on the association between dietary greenhouse gas emissions and cancer incidence. Both studies were part of EPIC, with one of them reporting on the overall study population [13] and the other presenting results for EPIC-Spain [8]. The analysis of EPIC-Spain yielded a non-statistically significant increased risk for cancer with every one unit increase in greenhouse gas emissions (HR: 1.031, 95% CI: 0.998–1.065; fully adjusted model), while the EPIC-wide analysis reported a statistically significantly increased risk for overall cancer incidence (HR _Q4__vs.__Q1_: 1.16, 95% CI: 1.10–1.22; fully adjusted model). The EPIC-wide analysis also reported a positive association between greenhouse gas emissions and risk for cancers of the breast, colorectum, stomach, and thyroid, among others (Table 1).

#### Land Use

The association between land use and cancer incidence was investigated in one study [13]. In this EPIC-wide investigation, higher land use was statistically significantly associated with increased risk of cancer (HR _Q4__vs.__Q1_: 1.13, 95% CI: 1.10–1.15; fully adjusted model). Positive associations were also reported for the risks of breast, colorectal, liver, stomach, and thyroid cancers.

#### Exposure to Pesticides or Organic Food Consumption

The association between exposure to pesticides or organic food consumption was investigated in four studies. Two studies used data from the NutriNet-Santé cohort. In a study focusing on fruit and vegetable consumption [14], higher consumption of either low or high pesticide residue fruit and vegetables was not associated with total cancer risk (HR _low-pesticide-residue_ _FVs_: 1.01, 95% CI: 0.99–1.02, HR _high-pesticide-residue_ _FVs_: 0.99, 95% CI: 0.97–1.01; per 1 serving/day increase in intake; fully adjusted model). Similar results were obtained when examining specific cancer sites (Table 1). In an analysis of the NutriNet-Santé cohort [15], different profiles of dietary pesticide exposure were investigated for their association with post-menopausal invasive breast cancer risk. Components highly correlated with chlorpyriphos, imazalil, malathion, profenofos, and thiabendazole were positively associated with post-menopausal invasive breast cancer risk (HR _Q5__vs.__Q1_: 1.77, 95% CI: 1.07–2.91; fully adjusted model). An inverse association was seen between components showing low correlations with synthetic pesticides and high correlation with the organic pesticide spinosad and post-menopausal invasive breast cancer risk (HR _Q5__vs.__Q1_: 0.59, 95% CI: 0.36–0.98, p-trend: 0.01; fully adjusted model).

When investigating the association between organic food consumption and cancer incidence, higher organic food scores were statistically significantly inversely associated with total cancer risk (HR _Q4__vs.__Q1_: 0.76, 95% CI: 0.64–0.90; fully adjusted model) [16]. Statistically significant inverse associations were also seen for post-menopausal breast cancer, Hodgkin lymphoma, and lymphomas (Table 1). In an analysis of the Million Women Study [17] focusing on self-reported frequency of organic food consumption, frequent organic food consumption (self-reported sometimes or usually/always vs. never organic food consumption) was positively associated with total cancer (RR _“Sometimes”_ _vs._ _“Never”_: 1.03, 95% CI: 1.01–1.04, RR _“Usually/always”_ _vs._ _“Never”_: 1.03, 95% CI: 1.00–1.06; fully adjusted model) as well as breast cancer risk (RR _“Sometimes”_ _vs._ _“Never”_: 1.07, 95% CI: 1.05–1.08, RR _“Usually/always”_ _vs._ _“Never”_: 1.09, 95% CI: 1.03–1.15; fully adjusted model). An inverse association was reported for non-Hodgkin lymphoma (RR _“Sometimes”_ _vs._ _“Never”_: 0.94, 95% CI: 0.90–0.99, RR _“Usually/always”_ _vs._ _“Never”_: 0.79, 95% CI: 0.67–0.94; fully adjusted model).

#### A Sustainability Index, Including Multiple Sustainability Indicators

The only study that combined multiple sustainability indicators in the form of an index was that of Seconda et al., analyzing data from the NutriNet-Santé cohort [18]. Their Sustainable Diet Index (SDI) included nutritional, environmental (synthetic pesticides, biodiversity preservation, greenhouse gas emissions, land occupation, and primary energy demand), and economic as well as food practice components. Higher SDI scores were associated with a statistically significantly lower risk for all cancers (HR_Q4__vs.__Q1_: 0.56, 95% CI: 0.41–0.77, *p*-trend: 0.0002; fully adjusted model).

### Cancer-Specific Mortality

#### Greenhouse Gas Emissions

The association between greenhouse gas emissions and cancer mortality was investigated in one study [13]. In this EPIC-wide investigation, higher greenhouse gas emissions were statistically significantly associated with increased cancer mortality (HR_Q4__vs.__Q1_: 1.16, 95% CI: 1.10–1.22; fully adjusted model).

#### Land Use

The association between land use and cancer mortality was investigated in one study [13]. In this EPIC-wide investigation, higher land use was statistically significantly associated with increased risk of cancer mortality (HR_Q4__vs.__Q1_: 1.21, 95% CI: 1.16–1.27; fully adjusted model).

#### Biodiversity

An EPIC-wide investigation explored the association between food biodiversity and cancer mortality [19]. An individual’s dietary food biodiversity was based on the absolute number of unique biological species reported in their diet. Higher dietary food biodiversity was inversely associated with cancer mortality (HR: 0.93, 95% CI: 0.92–0.95, per 10-species increment; fully adjusted model).

#### The EAT-Lancet Diet

One study reported on the association between the EAT-Lancet diet (i.e., the diet proposed by the EAT-Lancet Commission on Food, Planet, Health [20]) and cancer mortality [21]. Even though the sustainability of the diet was not explicitly assessed in the study, we allowed the inclusion of this study in the review on the basis that the EAT-Lancet diet per se was designed to not exceed the planetary resources. Higher adherence to the EAT-Lancet diet was inversely associated with cancer mortality (HR_Score__≥__23__vs.__≤__13_: 0.76, 95% CI: 0.63–0.92; fully adjusted model). In sex-specific analyses, the results remained statistically significant only for men.

#### Risk of Bias and Certainty of the Evidence Assessments

Our risk of bias assessment is summarized in Supplementary Fig. 1. Seven out of the nine identified studies showed a *serious* overall risk of bias, due to potential confounding (domain 1) and/or limitations of dietary assessments (domain 3). The certainty of the evidence was rated as *low* for three studies, essentially due to serious risk of bias (see Supplementary Table 2). Five studies were judged to have a *moderate* certainty of the evidence. For four of these studies, the certainty of the evidence assessment had initially been set to a *low* level due to serious risk of bias, but we agreed on upgrades to *moderate* certainty in view of dose–response gradients in the associations between dietary indices and cancer risks. We assigned a *high* certainty of the evidence to the study by Seconda et al. [18] despite moderate risk of bias, again due to a clear dose–response gradient of the reported associations between dietary index and cancer risk. It should be noted, however, that we did not apply two GRADE domains (inconsistency and publication bias) to our ratings given the lack of comparability of the dietary indices used in the identified studies. For the same reason, we could only assess single studies by the GRADE criteria (Supplementary Table 2).

## Discussion

In this systematic review, we summarized existing evidence on the association between sustainable diets and cancer. It is difficult to draw an overall conclusion due to the heterogeneity between the sustainability indicators and cancer types across the nine identified articles. However, a modest inverse association between sustainable diets and cancer incidence, or cancer mortality was observed in most studies, highlighting the dual benefit of adopting such diets for the environment and for human health.

The main challenge in our review was the lack of an agreed-upon definition of sustainability and the vastly different indicators that were assessed when characterizing the sustainability of a diet. Due to the heterogeneity of the sustainability indicators in the included studies, the limited number of studies identified in the literature, and the different approaches in estimating sustainability indicators, it was inappropriate to summarize the results with a common estimator using a meta-analysis approach.

The identified studies were characterized by moderate to serious risks of bias, mainly due to potential confounding, known limitations of questionnaire-based dietary assessments (i.e., selective reporting, recall bias, and, with regard to organic food consumption in two included studies, the use of non-validated questionnaire items) and a lack of information on changes in dietary preferences over time. Most studies had a “moderate” certainty of the evidence, in part despite “serious” risk of bias, given that associations between dietary indices and cancer risk or mortality in these studies showed a dose–response gradient. However, considering that the identified studies showed strong heterogeneity regarding dietary indices and cancer outcomes, our GRADE assessment of individual studies is clearly preliminary and needs to be interpreted with caution.

While we intentionally kept our search terms broad to ensure that we capture all available research on the topic, important sustainability indicators were not assessed in the included studies (e.g., blue water use, food waste). Another consideration when interpreting the results of our study is that all included studies came from western countries, with all but one paper being based on data from primarily Caucasian populations. Despite the lack of language, geographic, or time limitations in our search, we were unable to identify studies conducted in non-western countries. This is especially worrisome since based on recent projections, non-western countries that are currently experiencing an increase in the prevalence of established cancer risk factors (e.g., smoking, unhealthy diet) will face a 64–95% increase in the number of incident cancer cases diagnosed in 2040 [3]. Strikingly, in this context is the fact that diets lacking in fruit, vegetables and whole grains are particularly strong drivers of a greater burden of disease in countries with a lower social development index [22] according to the latest report of the Global Burden of Disease Study.

Due to the inherently dynamic nature and complex relationships in food systems, it is unlikely that a “one size fits all” solution will be able to address the multiple challenges they face. Priorities should be set to ensure equitable access to high-quality food, focusing on those individuals who are not only most vulnerable to changes in the food system but who also face the highest consequences of climate change and malnutrition [23]. Multi-sectorial, multidisciplinary, concentrated actions will be needed to simultaneously reduce the risk of all forms of malnutrition that have common drivers (e.g., biology, epigenetics of early life nutrition, socioeconomic factors) [24] and non-communicable diseases and address the ongoing climate crisis.

Putting the onus, and all efforts for change, on individual dietary consumption and individual responsibility, is unlikely to effectively change food systems, improve diet quality and reduce food insecurity and the health consequences of all forms of malnutrition. A wide range of interconnected efforts targeting factors such as food production, food processing, and distribution, campaigns, food labeling, and food pricing strategies [22], which have been proven cost-effective, are needed to effectively introduce change.

The results of this study highlight the urgent need for high-quality research in the field of sustainable diets, with a particular emphasis on studies conducted outside the European/western context. Additionally, they emphasize the need for a standardized approach when assessing sustainability, making sure all important aspects are included in all indices or sustainability assessment indicators. A more streamlined approach and the use of a universally agreed-upon definition on what constitutes a sustainable diet will allow for comparability between future studies.

Strengths of our study are the extensive systematic search, with broad search terms, in two large databases to increase the possibility of identifying all relevant articles. To the best of our knowledge, this is the first systematic overview of the available literature on sustainable diets and cancer. Additionally, all identified articles were screened and assessed by two researchers, including the risk of bias assessment. Limitations of the study include the self-reported nature of dietary assessment in the included studies that could have led to misclassification of actual behaviors. Due to the heterogeneity of the extracted data, it was not possible to conduct a meta-analysis, and our assessment of the certainty of the evidence remains preliminary for the same reason. Finally, since most studies were conducted in Europe, results might primarily be applicable to European/western populations, limiting the generalizability of our results.

## Conclusions

In conclusion, while it is challenging to draw an overall conclusion due to the heterogeneity in the sustainability indicators used, a modest inverse association between sustainable diets and cancer incidence, or cancer mortality, was observed in most studies. By adopting sustainable diets, we might experience co-benefits for both the environment and human health.

## Supplementary Information

Below is the link to the electronic supplementary material.

Supplementary file1 (DOCX 108 KB)

Supplementary file2 (DOCX 12 KB)
