# Supplementary material for: Sustainable Diets and Cancer: a Systematic Review
Source: Curr Nutr Rep. 2022 Nov 21;11(4):742–52. doi: 10.1007/s13668-022-00442-z (PMC9750932; doi:10.1007/s13668-022-00442-z)
Supplement: Supplementary file 1 — Supplementary file1 (DOCX 108 KB) [file 13668_2022_442_MOESM1_ESM.docx]

Supplementary Table 1. Study characteristics and description of the sustainability indicators and their assessment

| Author | Publication Year | Country | Study name | Year(s) of baseline data collection | Sustainability Indicator | Sustainability assessment | Dietary assessment (if necessary to compute the sustainability indicator) |
| --- | --- | --- | --- | --- | --- | --- | --- |
| González | 2020 | Spain | EPIC-Spain | Mostly between 1992 and 1998 | Dietary GHG emissions | Data on food GHG emission in kgCO_2_eq/kg for 57 food items of EPIC-Spain were obtained from a systematic review from LCA studies (1718 GWP values for 168 varieties of fresh food products; most of the food GWP values collected in this database comes from Europe, UK and EEUU, but there are also values from Asia, South America and Africa). The GHG emission mean value for 133 food items from this systematic review (expressed in kgCO_2_eq/kg of food) was applied to the same food items of the 240 food list of EPIC-Spain. Finally, GHG emission value of 57 food items from EPIC, representing 68% of the mean of total calories intake of the participants, were considered in the analysis. | Each participant’s usual food intake was obtained through individual interviews at recruitment by using a validated electronic dietary history questionnaire. The EPIC-Spain dietary questionnaire included a list of 662 registered foods that were grouped and reduced to 240 food items used in EPIC Europe studies. |
| Hanley-Cook | 2021 | 9 European countries | EPIC | 1992 to 2000 | Individual diet food biodiversity (dietary species richness) based on the absolute number of unique biological species | Food biodiversity in an individual’s diet was calculated based on the absolute number of unique biological species in each (composite) food, drink, and recipe, using the European Food Safety Authority’s FoodEx2 food classification and description system in combination with the detailed EPIC food classification system (NCLASS). Food items consumed “never or less than once per month” (on average) were recalled under one category; accordingly, these species did not count toward dietary species richness (DSR). Moreover, quantities (g/day) were disregarded for overall DSR computation, since the interest is the sum of distinct species consumed per year (i.e., DQs recalled dietary intake over the preceding 12 months). Furthermore, although a species can be consumed multiple times per year, potentially from diverse functional food groups (e.g., chicken meat and eggs, which are nutritionally disparate), through a “biodiversity conservation” lens, it contributes only one species to an individual’s DSR in all scenarios (taxonomically identical: Gallus gallus). | Usual dietary intake was assessed for each individual at recruitment using country- or center-specific validated DQs developed to capture the geographical specificity of an individual’s diet over the preceding year. |
| Laine | 2021 | 10 European countries | EPIC | 1991 to 2000 | Greenhouse gas emissions, land use | Greenhouse gas emissions and land use were estimated from detailed standardized country-specific dietary questionnaires, with the SHARP-Indicators Database, a European-wide database for estimating environmental impacts of food production, packaging, transport, and home preparation. The total food list for EPIC comprised 11 858 food items. Specific food items were matched between the EPIC database and the SHARP database, based on their FoodEx2 code from the Exposure Hierarchy of the European Food Safety Authority. Greenhouse gas emissions were expressed as kg CO_2_ equivalents per kg food per day and land use as m² per year per kg food per day. | Usual dietary intake was assessed for each individual at recruitment using country- or center-specific validated DQs developed to capture the geographical specificity of an individual’s diet over the preceding year. |
| Stubbendorff | 2021 | Sweden | Malmö Diet and Cancer Study | 1991 to 1996 | EAT-Lancet diet score (range 0 (nonadherence) to 42 points (perfect adherence = 14 × 3 points)) | The EAT-Lancet diet consists of food components for which defined target intake levels and reference intervals (ranges) are suggested. Food components were classified as either “emphasized foods” or “limited foods” based on previous descriptions of the EAT-Lancet diet. Emphasized food components were vegetables, fruits, unsaturated oils, legumes, whole grains, nuts, and fish. Food components classified as limited were beef and lamb, pork, poultry, eggs, dairy, potatoes, and added sugar. Dietary intakes were evaluated based on reported amounts in grams per day in uncooked weight, which is in line with how the target intake levels are expressed in the EAT-Lancet diet. In this study, the index consists of the 14 food components, with a possible range of 0–3 points for each component; 0 points indicates low adherence to the target for the food component in the EAT-Lancet diet and 3 points indicates high adherence. | Dietary intake was assessed using a validated, modified diet history method consisting of 3 parts: 1) a 7-day (consecutive days) food diary covering meals that vary from day to day (primarily lunch and dinner), cold beverages (including alcoholic beverages), and dietary supplements; 2) a 168-item FFQ covering consumption frequencies and portion sizes of food regularly consumed, such as breakfasts and snacks, in the past 12 months and not covered by the food diary; and 3) a 60-minute interview conducted to ask for cooking methods and usual portion sizes and to check for overlap between intakes reported by the 7-day food diary and the FFQ. |
| Sandoval-Insausti | 2021 | USA | Nurses’ Health Study, Nurses’ Health Study II and Health Professionals Follow-up Study | 1998 and 1999 | Pesticide Residue Burden as a score | The Pesticide Residue Burden Score (PRBS) is a scoring system that allows for the assessment of pesticide residue content of FVs using surveillance data collected as part of the Pesticide Data Program (PDP). This score has been validated against urinary pesticide metabolites in other cohorts. FFQ data from 1998 (NHS/HPFS) and 1999 (NHSII) were matched with PDP data from 1996 to 1999; FFQ data from 2002 (NHS/HPFS) and 2003 (NHSII) were matched with PDP data from 2000 to 2003; etc. Each fruit or vegetable was categorized according to 3 PDP estimations: the percentage of samples that presented detectable pesticide residues, the percentage of samples with pesticide residues above the tolerances levels, and, the percentage of samples which contained three or more individual detectable pesticides. For each contamination measure, a score of 0 (lowest), 1, or 2 (highest) was given to each FV based on the tertile distribution of each measure; those component scores were summed to calculate the PRBS, which could thus range from 0 to 6. For each period, FVs with a PRBS ≥ 4 were considered as high-pesticide-residue FVs, those with a PRBS < 4 as low-pesticide-residue FVs, and those without contamination information in a specific period as undetermined-pesticide-residue FVs. Finally, intakes of high-, low-, and undetermined-pesticide-residue FVs were summed for each participant. | Every four years, dietary information has been assessed with a validated FFQ with 131 food items. To maximize the overlap of available data from the FFQs and the PDP, analysis baseline was set to 1998 for NHS and HPFS and 1999 for NHSII. |
| Rebouillat | 2021 | France | NutriNet-Santé | 2014 | Dietary pesticide exposure profiles (based on daily intake of 25 commonly used active pesticides) as four NMF components divided into quintiles: NMF Component 1: highly correlated with chlorpyriphos, imazalil, malathion, profenofos and thiabendazole NMF Component 2: highly correlated pesticides were azoxystrobin, boscalid, cyprodinil, difenoconazole, fenhexamid, iprodione, tebuconazole and lambda-cyhalothrin NMF Component 3: low correlations with synthetic pesticides and high correlation with organic pesticide spinosad NMF Component 4: high correlations with acetamiprid, carbendazim, chlorpyrifos, cypermethrin and dimethoate/omethoate | Dietary pesticide exposure was estimated by combining dietary intakes of each adult with pesticide-residue concentration values in foods using contamination data from *Chemisches und Veterinäruntersuchungsamt* (CVUA) Stuttgart, a EU reference laboratory for pesticides. The database comprised contamination data for conventional and organic-food products. 25 commonly used pesticides were selected among components available in this database, given both their frequency of detection above the maximum residue levels when sufficient data were available and their frequency above the acceptable daily intake otherwise. Pesticides commonly used in organic agricultural systems (e.g. natural pyrethrins, spinosad) were also selected. These criteria made it possible to take into account a broad spectrum of classes of pesticides. The 264 Org-FFQ items were decomposed into 442 ingredients (comprising 5% of at least one food item). Animal-based ingredients were excluded, as CVUA encompassed plant-based ingredients only. The resulting 180 plant ingredients were matched to the CVUA database and then were attributed a contamination value in organic and conventional farming modes (as the mean of the corresponding data point). For each ingredient/pesticide pair in conventional and organic farming, a frequency of detection and a frequency of quantification were determined.  Food-consumption data from NutriNet-Santé referred to edible foods (bone-free, peeled or cooked products); edibility and cooking factors were allocated to each ingredient when necessary. Cooking or peeling effects on pesticide-residue levels were not accounted for, as dilution factors are not available for all food/pesticide couples. For each pesticide, the estimated daily intake (in µg/kg of weight/day) under both lower and upper-bound scenarios was calculated using methods recommended by the European Food and Safety Authority and the World Health Organization. The lower-bound (optimistic) scenario was used for this work, as it was more in line with the available literature comparing both production systems. | An organic food semi-quantitative frequency questionnaire (Org-FFQ), based on a previously validated FFQ comprising 264 items (food and beverages) was used to estimate organic and conventional food consumption. Participants reported, for each item, their consumption frequency, the portion size consumed (described as color photographs or typical household measurements), and the frequency of consumption of each item in its organic form (referring to French/European Union-certified organic labeling) through 5 modalities: never, rarely, half of the time, often and always. |
| Seconda | 2020 | France | NutriNet-Santé | 2014 | SDI, based on nutritional, environmental (indicators include: synthetic pesticides, biodiversity preservation, greenhouse gas emissions, land occupation and primary energy demand), economic and food practices sub-indexes. | A database gathering three environmental indicators, greenhouse gas emissions (in kg CO_2_eq), primary energy consumption (in MJ), and land occupation (in m²) related to raw food production and taking into account the mode of food production (organic vs. conventional) was developed using the DIALECTE database and completed with published literature data. Conversion factors (cooking and preparation factors) were used to assign environmental indicators for each item from farm to fork (i.e., from raw agricultural products to food consumption). GHGEs, primary energy consumption and land occupation of each individual diet was calculated by multiplying each indicator of food by the quantity of consumed food. As different indicators reflect different pressures and impacts on the environment, an overall score has been developed, and named the ReCiPe method. For food products, a partial score, the pReCiPe, including the GHGEs, land occupation, and energy demand was also proposed [pReCiPe = (0.0459*greenhouse gas emissions (in kg CO_2_eq) + 0.0025*primary energy consumption (in MJ) + 0.0439*land occupation (in m²))/ total diet weight without water]. | An organic food semi-quantitative frequency questionnaire (Org-FFQ), based on a previously validated FFQ comprising 264 items (food and beverages) [32] was emailed to participants in June to December 2014, to estimate organic and conventional food consumption. Participants reported, for each item, their consumption frequency, the portion size consumed (described as color photographs or typical household measurements), and the frequency of consumption of each item in its organic form (referring to French/European Union-certified organic labeling) through 5 modalities: never, rarely, half of the time, often and always. |
| Baudry | 2018 | France | NutriNet-Santé | 2009 | OFS | Volunteers were asked to provide information on their consumption frequency of 16 labeled organic products (fruits; vegetables; soy-based products; dairy products; meat and fish; eggs; grains and legumes; bread and cereals; flour; vegetable oils and condiments; ready-to-eat meals; coffee, tea, and herbal tea; wine; biscuits, chocolate, sugar, and marmalade; other foods; and dietary supplements). Consumption frequencies of organic foods were reported using the following 8 modalities: (1) most of the time, (2) occasionally, (3) never (“too expensive”), (4) never (“product not available”), (5) never (“I’m not interested in organic products”), (6) never (“I avoid such products”), (7) never (“for no specific reason”), and (8) “I don’t know.” For each product, 2 points were allocated for “most of the time” and 1 point for “occasionally” (and 0 otherwise). The 16 components were summed to provide an OFS (range, 0-32 points). |  |
| Bradbury | 2014 | United Kingdom | Million Women Study | 2002 | Frequency of consumption of OF | At the 3-year survey, completed in 2002 on average, women were asked ‘Do you eat organic food?’ with four possible categorical responses: ‘never, sometimes, usually, and always.’ |  |

Abbreviations: BMI: Body Mass Index; CI: Confidence Interval; CNS: Central Nervous System; DQ: Dietary questionnaires; EPIC: European Prospective Investigation into Cancer and Nutrition; FFQ: food frequency questionnaire; FV: fruit and vegetables; GHG: Greenhouse gas; GWP: Global warming potential; HR: Hazard Ratio; LCA: Life cycle assessment; NMF: Non-negative Matrix Factorization; OF: Organic Food; OFS: organic food score; RR: Relative Risk; SDI: Sustainable Diet Index.

Supplementary Table 2. GRADE Assessment, individual domains and overall^1^

|  | ***Downgrading*** | | | | | ***Upgrading*** | ***Overall Assessment*** |
| --- | --- | --- | --- | --- | --- | --- | --- |
|  | *Risk of Bias* | *Inconsistency* | *Imprecision* | *Indirectness* | *Publication Bias* | *Effect size, dose-response gradient, effect of plausible residual confounding* |  |
| González et al., 2020 | Downgraded by two levels, serious risk of bias | Not applicable, lack of comparable studies | Not downgraded given the large sample size | Not downgraded, population-based study design | Not applicable | No upgrade | ⨁⨁◯◯  LOW |
| Laine et al., 2021 | Downgraded by two levels, serious risk of bias | Not applicable, lack of comparable studies | Not downgraded given the large sample size | Not downgraded, population-based study design | Not applicable | Upgraded by one level, dose-response gradient | ⨁⨁⨁◯  MODERATE |
| Sandoval-Insausti et al., 2021 | Downgraded by two levels, serious risk of bias | Not applicable, lack of comparable studies | Not downgraded given the large sample size | Not downgraded, population-based study design | Not applicable | No upgrade | ⨁⨁◯◯  LOW |
| Rebouillat et al., 2021 | Downgraded by one level, moderate risk of bias | Not applicable, lack of comparable studies | Not downgraded given the large sample size | Not downgraded, population-based study design | Not applicable | No upgrade | ⨁⨁⨁◯  MODERATE |
| Seconda et al., 2021 | Downgraded by one level, moderate risk of bias | Not applicable, lack of comparable studies | Not downgraded given the large sample size | Not downgraded, population-based study design | Not applicable | Upgraded by one level, dose-response gradient | ⨁⨁⨁⨁  HIGH |
| Baudry et al., 2018 | Downgraded by two levels, serious risk of bias | Not applicable, lack of comparable studies | Not downgraded given the large sample size | Not downgraded, population-based study design | Not applicable | Upgraded by one level, dose-response gradient | ⨁⨁⨁◯  MODERATE |
| Bradbury et al., 2014 | Downgraded by two levels, serious risk of bias | Not applicable, lack of comparable studies | Not downgraded given the large sample size | Not downgraded, population-based study design | Not applicable | No upgrade | ⨁⨁◯◯  LOW |
| Hanley-Cook et al., 2021 | Downgraded by two levels, serious risk of bias | Not applicable, lack of comparable studies | Not downgraded given the large sample size | Not downgraded, population-based study design | Not applicable | Upgraded by one level, dose-response gradient | ⨁⨁⨁◯  MODERATE |
| Stubbendorff et al., 2021 | Downgraded by two levels, serious risk of bias | Not applicable, lack of comparable studies | Not downgraded given the large sample size | Not downgraded, population-based study design | Not applicable | Upgraded by one level, dose-response gradient | ⨁⨁⨁◯  MODERATE |
| ^1^Assessment of reported results based on the primary endpoints of the studies. | | | | | | | |


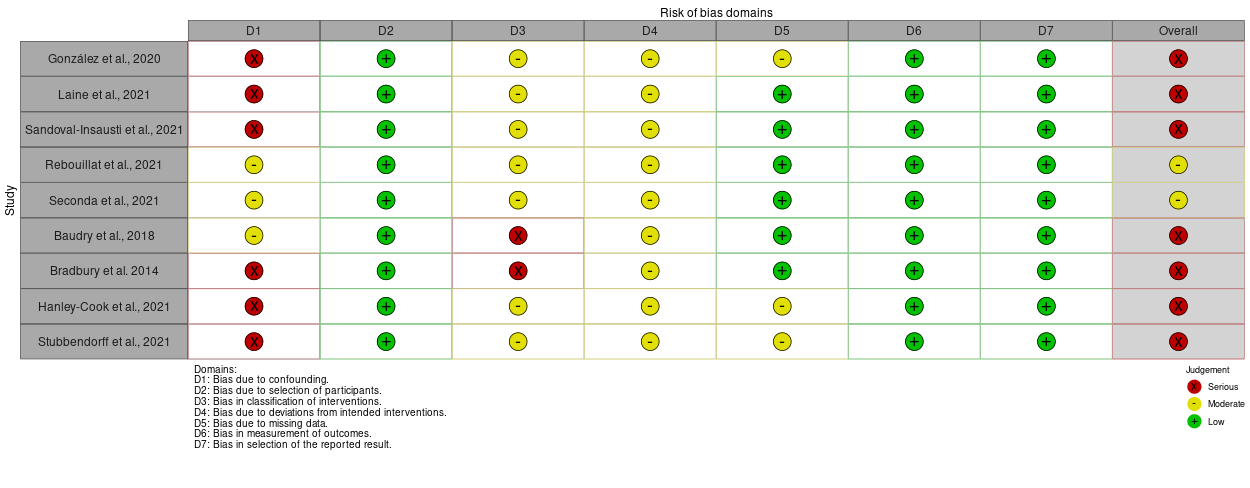


**Supplementary Figure 1.** Risk of Bias Assessment (RoB)

Regarding confounding (RoB domain **D1**), we first assigned ‘moderate’ RoB to all studies due to potential confounding. Moreover, in six out of the nine included studies there was no statistical adjustment for at least one of the confounders that we had specified before our RoB assessment (age, sex, education level, smoking status, alcohol consumption, physical activity, body fatness, height, reproductive factors, and energy intake). Thus, we judged that there was ‘serious’ a RoB in these studies. We identified no RoB related to participant selection (**D2**). While the included cohorts may be affected by ‘healthy cohort bias’, it is conceivable that a selection of more health-conscious participants may lead to an underestimation of relative risks. The classification of the exposures (**D3**) was mostly based on validated dietary assessment tools and databases for environmental indicators. Given known methodological issues with dietary assessment tools (e.g., selective reporting, recall bias), we assigned a moderate RoB to most studies. In two studies on organic food (Baudry et al. 2018 and Bradbury et al. 2014), the food frequency questionnaires were not validated for assessing organic food consumption, with serious RoB. Moderate RoB was assigned to all included studies regarding deviations in exposures over time (**D4**), as changes in dietary preferences were not assessed or accounted for in statistical analyses. Again, however, it can be assumed that changes in dietary preferences over time would attenuate true effects rather than reverse them. Information on missing data (**D5**) was in part not provided for three studies, which is why moderate RoB was assigned. The outcome assessment (**D6**) was adequate in all studies. There was no indication for selective reporting (**D7**).

**References**

Baudry J, Assmann KE, Touvier M, et al (2018) Association of Frequency of Organic Food Consumption With Cancer Risk: Findings From the NutriNet-Santé Prospective Cohort Study. JAMA Intern Med 178:1597–1606. https://doi.org/10.1001/jamainternmed.2018.4357

Bradbury KE, Balkwill A, Spencer EA, et al (2014) Organic food consumption and the incidence of cancer in a large prospective study of women in the United Kingdom. Br J Cancer 110:2321–2326. https://doi.org/10.1038/bjc.2014.148

González CA, Bonet C, de Pablo M, et al (2021) Greenhouse gases emissions from the diet and risk of death and chronic diseases in the EPIC Spain cohort. Eur J Public Health 31:130–135. https://doi.org/10.1093/eurpub/ckaa167

Hanley-Cook GT, Huybrechts I, Biessy C, et al (2021) Food biodiversity and total and cause-specific mortality in 9 European countries: An analysis of a prospective cohort study. PLoS Med 18:e1003834. https://doi.org/10.1371/journal.pmed.1003834

Laine JE, Huybrechts I, Gunter MJ, et al (2021) Co-benefits from sustainable dietary shifts for population and environmental health: an assessment from a large European cohort study. Lancet Planet Heal 5:e786–e796. https://doi.org/10.1016/S2542-5196(21)00250-3

Rebouillat P, Vidal R, Cravedi J-P, et al (2021) Prospective association between dietary pesticide exposure profiles and postmenopausal breast-cancer risk in the NutriNet-Santé cohort. Int J Epidemiol 50:1184–1198. https://doi.org/10.1093/ije/dyab015

Sandoval-Insausti H, Chiu Y-H, Lee DH, et al (2021) Intake of fruits and vegetables by pesticide residue status in relation to cancer risk. Environ Int 156:106744. https://doi.org/10.1016/j.envint.2021.106744

Seconda L, Baudry J, Allès B, et al (2020) Prospective associations between sustainable dietary pattern assessed with the Sustainable Diet Index (SDI) and risk of cancer and cardiovascular diseases in the French NutriNet-Santé cohort. Eur J Epidemiol 35:471–481. https://doi.org/10.1007/s10654-020-00619-2

Stubbendorff A, Sonestedt E, Ramne S, et al (2022) Development of an EAT-Lancet index and its relation to mortality in a Swedish population. Am J Clin Nutr 115:705–716. https://doi.org/10.1093/ajcn/nqab369
