# Supplementary material for: Sustainable Diets and Cancer: a Systematic Review
Source: Curr Nutr Rep. 2022 Nov 21;11(4):742–52. doi: 10.1007/s13668-022-00442-z (PMC9750932; doi:10.1007/s13668-022-00442-z)
Supplement: Supplementary file 2 — Supplementary file2 (DOCX 12 KB) [file 13668_2022_442_MOESM2_ESM.docx]

Supplementary Information

PubMed search: ("cancer"[All Fields] OR "neoplasms"[MeSH Terms]) AND ("planetary health"[All Fields] OR "sustainable diet"[All Fields] OR "food biodiversity"[All Fields] OR "greenhouse gas"[All Fields] OR "greenhouse gasses"[All Fields] OR "GHG"[All Fields] OR "environmental footprint"[All Fields] OR "sustainable food"[All Fields] OR "organic food"[All Fields])

EMBASE search: ('cancer':af OR 'neoplasms'/exp OR 'neoplasms') AND ('planetary health'/exp OR 'planetary health' OR 'sustainable diet' OR 'food biodiversity' OR 'greenhouse gas'/exp OR 'greenhouse gas' OR 'greenhouse gasses' OR 'ghg' OR 'environmental footprint'/exp OR 'environmental footprint' OR 'sustainable food' OR 'organic food'/exp OR 'organic food')
